# Hematopoietic Stem and Progenitor Cells Acquire Distinct DNA-Hypermethylation During *in vitro* Culture

**DOI:** 10.1038/srep03372

**Published:** 2013-11-28

**Authors:** Carola Ingrid Weidner, Thomas Walenda, Qiong Lin, Monika Martina Wölfler, Bernd Denecke, Ivan Gesteira Costa, Martin Zenke, Wolfgang Wagner

**Affiliations:** 1 Helmholtz-Institute for Biomedical Engineering, RWTH University Medical School, Aachen, Germany; 2 Institute for Biomedical Technology – Cell Biology, RWTH University Medical School, Aachen, Germany; 3 Department of Obstetrics and Gynecology, RWTH University Medical School, Aachen, Germany; 4 Interdisciplinary Centre for Clinical Research (IZKF), RWTH University Medical School, Aachen, Germany

## Abstract

Hematopoietic stem and progenitor cells (HPCs) can be maintained *in* *vitro*, but the vast majority of their progeny loses stemness during culture. In this study, we compared DNA-methylation (DNAm) profiles of freshly isolated and culture-expanded HPCs. Culture conditions of CD34^+^ cells - either with or without mesenchymal stromal cells (MSCs) - had relatively little impact on DNAm, although proliferation is greatly increased by stromal support. However, all cultured HPCs - even those which remained CD34^+^ - acquired significant DNA-hypermethylation. DNA-hypermethylation occurred particularly in up-stream promoter regions, shore-regions of CpG islands, binding sites for PU.1, HOXA5 and RUNX1, and it was reflected in differential gene expression and variant transcripts of *DNMT3A*. Low concentrations of DNAm inhibitors slightly increased the frequency of colony-forming unit initiating cells. Our results demonstrate that HPCs acquire DNA-hypermethylation at specific sites in the genome which is relevant for the rapid loss of stemness during *in vitro* manipulation.

Culture expansion of hematopoietic stem and progenitor cells (HPCs) remains one of the major challenges in stem cell biology. This is of particular relevance for umbilical cord blood (CB) where the number of available stem cells is limited and often insufficient for transplantation of adult patients^1^. It is also crucial for *in vitro* manipulation of HPCs in gene therapy approaches^2^. Proliferation of HPCs can be stimulated *in vitro* but it is usually associated with loss of stem cell properties. Culture conditions can be optimized by combinations of growth factors^3^, small synthetic chemicals^4^ or specific proteins including Notch-ligands^5^. It has also been shown that co-culture with mesenchymal stromal cells (MSCs) mimics components of the hematopoietic niche and thereby supports maintenance of primitive HPCs *in vitro*^6^,^7^,^8^. However, all of these approaches have in common that the vast majority of the proliferating progeny loses regenerative potential within few days. A better understanding of molecular changes during culture might facilitate controlled *in vitro* expansion of the multipotent subset.

DNA-methylation (DNAm) of CpG dinucleotides is a key epigenetic modification. Upon cell division, the DNAm pattern is maintained on the newly synthesized DNA strand particularly by DNA methytransferase 1 (DNMT1), whereas DNMT3A and DNMT3B act as *de novo* methyltransferases and modify unmethylated CpG sites during differentiation^9^. Conversely, active demethylation may be promoted by methyl-CpG binding proteins or *via* a hydroxymethylate intermediate step^10^. DNAm plays a central role in normal hematopoietic development and consequently, it might also be relevant for the rapid loss of stem cell activity during *in vitro* culture^11^.

In this study, we analyzed if the DNAm pattern of CB derived CD34^+^ HPCs is modified during culture expansion either with or without stromal support. DNAm profiles were determined using a novel Infinium HumanMethylation450 platform which assays more than 480,000 CpG sites at single base resolution (covering 99% of RefSeq genes and 96% of CpG islands)^12^. We demonstrate that culture expansion induces specific hypermethylation in relevant hematopoietic genes.

## Results

### Expansion of hematopoietic progenitor cells affects DNAm profiles

**Figure 1:**
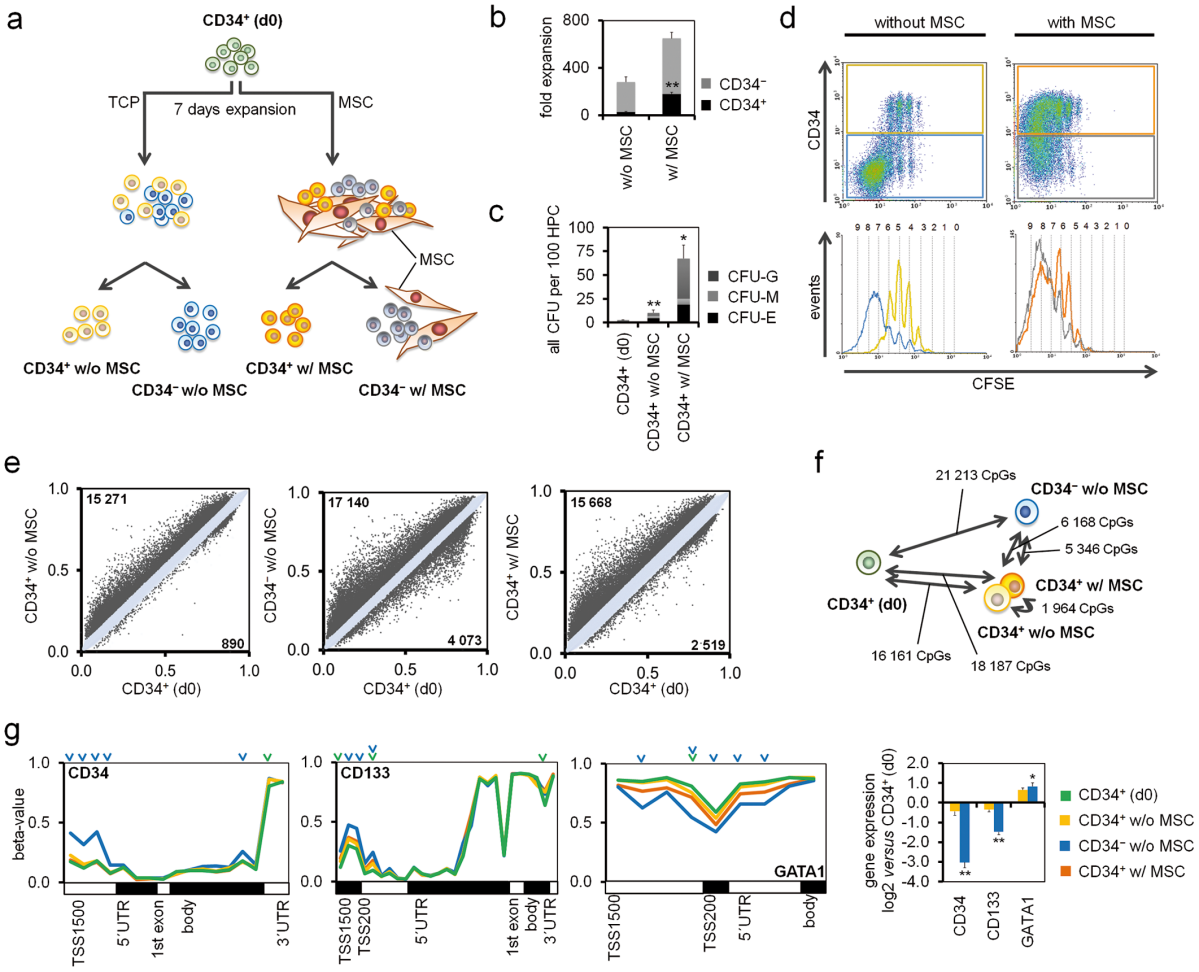
Culture expansion of CD34^+^ cells affects DNAm. (a) Experimental design. CD34^+^ cells (d0) were cultured in parallel either with or without mesenchymal stromal cells (MSCs). After one week, HPCs were separated into CD34^+^ and CD34^−^ fractions. (b) MSCs significantly stimulated proliferation and maintenance of CD34 expression. (c) Colony-forming unit (CFU)-frequency of freshly isolated and expanded HPCs. CFU-initiating cells were particularly increased by stromal support of MSCs (n = 3; *p ≤ 0.05; **p ≤ 0.01). (d) HPCs were stained with carboxyfluorescein diacetate N-succinimidyl ester (CFSE) before culture expansion. As indicated in the histograms, each peak is associated with a unique number of cell divisions. MSCs enhance proliferation of HPCs and maintain CD34 expression for more cell divisions. (e) Scatter plots demonstrate significant DNAm changes upon culture expansion (adjusted p < 0.05). In comparison to freshly isolated CD34^+^ (d0) cells, all expanded fractions (CD34^+^ w/o MSC, CD34^−^ w/o MSC or CD34^+^ w/MSC) revealed particularly hypermethylation at specific CpG sites. (f) Number of differentially methylated CpG sites (hyper- and hypomethylated) between the different subsets. DNAm profiles of CD34^+^ w/o MSC and CD34^+^ w/MSC were closely related. (g) DNAm of CpG sites is exemplarily depicted for the genes *CD34*, *CD133* and *GATA1*. Promoter regions of *CD34* and *CD133* become higher methylated in the CD34^−^ progeny. Conversely, *GATA1* is rather hypomethylated and up-regulated on gene expression level. Significantly changed CpG sites (adjusted p < 0.05) are indicated by arrowheads (*vs.* CD34^+^ (d0) depicted in green; *vs.* CD34^−^ w/o MSC in blue; *p ≤ 0.05; **p ≤ 0.01). See also Supplementary Fig. S1 and S2.

CD34^+^ cells were cultured for seven days either on tissue culture plastic (TCP) or in co-culture with MSCs (Fig. 1a). Notably, the CD34^+^ fraction is heterogeneous and only a small subset resembles hematopoietic stem cells (HSCs). Stromal support greatly increased cellular proliferation, the percentage of CD34^+^ cells, and colony forming unit (CFU)-frequency (Fig. 1b–d). We have previously shown that these culture conditions expand CD34^+^ cells *in vitro*, and that NOD/SCID mice repopulating cells were particularly maintained with stromal support^13^. The cellular size of HPCs increased during culture, they became more elongated and overall CD34 and CD133 expression decreased, whereas differentiation markers were upregulated (Supplementary Fig. S1). Taken together, stromal support facilitates expansion of HPCs, but the vast majority of the progeny further proceeds towards differentiation.

We expected that DNAm changes were particularly acquired in the faster proliferating subset which loses CD34 expression. To test this thesis, we separated cultured HPCs into a CD34^+^ and a CD34^−^ fraction and compared their DNAm profiles with freshly isolated HPCs. Furthermore, HPCs were co-cultured with MSCs to estimate the impact of stromal support on DNAm profiles. CD34^−^ w/MSCs were excluded due to contamination of MSCs. The overall DNAm level was hardly affected by culture expansion (Supplementary Fig. S2a,b), but there were many significant changes at specific CpG sites (adjusted p-value < 0.05): CD34^+^ cells without stromal support (CD34^+^ w/o MSC) accumulated 15,271 hypermethylated and 890 hypomethylated CpG sites; CD34^−^ w/o MSC cells showed 17,140 hypermethylated and 4,073 CpG hypomethylated sites; and the CD34^+^ w/MSC fraction revealed 15,668 hypermethylated and 2,519 hypomethylated CpG sites (Fig. 1e). Thus, *in vitro* culture of HPCs results predominantly in hypermethylation of specific CpG sites.

Unexpectedly, DNAm profiles of culture expanded CD34^+^ *versus* CD34^−^ subsets revealed fewer differences: 4,304 CpG sites were higher methylated in CD34^+^ w/o MSC, whereas 1,864 CpG sites were higher methylated in CD34^−^ w/o MSC (Fig. 1f, Supplementary Fig. S2c). We reasoned that these DNAm changes might reflect differentiation of the CD34^−^ subset. In fact, some of the most significant hypermethylation in CD34^−^ w/o MSC was observed in *CD34* (adjusted p = 0.0003) and *CD133* (p = 0.005), whereas several genes involved in hematopoietic differentiation, such as GATA binding protein 1 (*GATA1*; p = 0.0007), were rather hypomethylated (Fig. 1g). To validate DNAm changes we have exemplarily analyzed the differentially methylated region in the promoter region of the gene *CD34* using bisulfite pyrosequencing in independent samples. As observed by the HumanMethylation450 platform the CD34^−^ cell fraction revealed significant hypermethylation (Supplementary Fig. S3a).

Stromal support had even less impact on DNAm profiles: comparison of CD34^+^ w/o MSC *versus* CD34^+^ w/MSC revealed only 848 hypermethylated and 1,116 CpGs hypomethylated CpG sites (Supplementary Fig. S2c). Thus, co-culture with MSCs does not prevent *in vitro* culture associated DNAm changes, but it seems to shift this process to higher cell division numbers.

### DNAm changes are enriched in genes involved in hematopoietic development

Subsequently, we focused on the CpG sites which were differentially methylated upon culture expansion. These modifications might be related to senescence. Long term culture of other cell types, such as MSCs, has been associated with specific senescence-associated DNAm (SA-DNAm) changes, which can be used for monitoring of senescence^14^,^15^. However, DNAm changes upon culture of HPCs revealed only a very moderate association with SA-DNAm changes indicating that they were not related to replicative senescence (Supplementary Fig. S2d).

**Figure 2:**
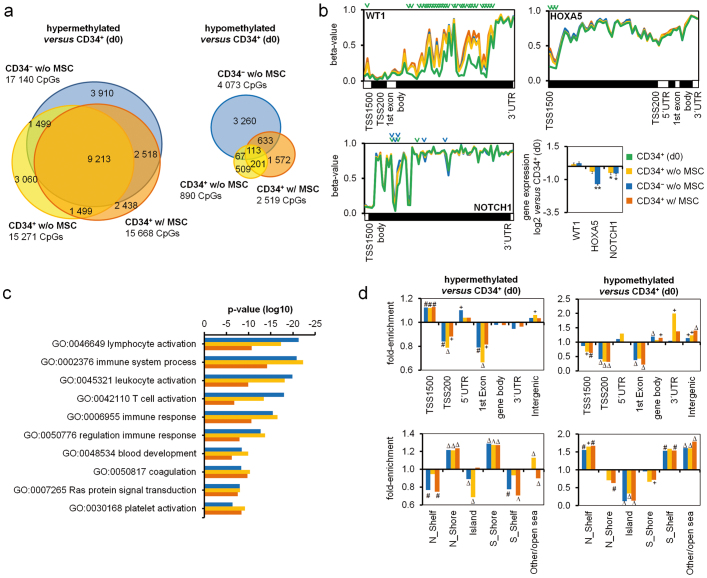
Distribution of differentially methylated CpG sites. (a) Overlap of hypermethylated and hypomethylated CpG sites in the expanded cell fractions. (b) For the genes *WT1*, *HOXA5* and *NOTCH1* site specific DNAm changes (arrowheads indicate significant CpG sites in comparison to either CD34^+^ (d0) [green] or CD34^−^ w/o MSC [blue]) and gene expression changes are exemplarily depicted (*p ≤ 0.05; **p ≤ 0.01). (c) Gene Ontology analysis revealed most significant enrichment of DNAm changes in genes related to categories of hematopoietic activation or immune processes (same colour code as in B). (d) Enrichment of DNAm changes in relation to gene regions or CpG islands. Hypermethylation was enriched in TSS1500 and shore regions, whereas hypomethylation was enriched in 3′UTR, intergenic areas and shelf regions. (hypergeometric distribution: +p < 10^−5^; #p < 10^−10^; Δp < 10^−15^).

All expanded cell fractions (CD34^+^ w/o MSC, CD34^−^ w/o MSC and CD34^+^ w/MSC) revealed a remarkable overlap in hypermethylation (Fig. 2a). Among these was the Wilms tumor 1 gene (*WT1*; P < 10^−123^); *NOTCH1* (p < 10^−25^), a known modulator of lineage-specific events in hematopoiesis^16^; and various genes of the homeobox gene cluster A (particularly *HOXA5*: p < 10^−35^; Fig. 2b, Supplementary Fig. S4a). Hypermethylation was also observed in the HOXB cluster (particularly *HOXB3*; p < 10^−17^), but not in *HOXB4* which has previously been implicated in *in vitro* expansion of HPCs (Supplementary Fig. S4b)^17^,^18^. Other relevant genes with hypermethylated CpG sites include the myeloid translocation gene 16 (*MTG16*; also known as *CBFA2T3*; p < 10^−150^) that has been implicated in the maintenance of stem cell quiescence; the retinoid X receptor alpha (*RXRA*; p < 10^−91^), whose down-regulation is essential for neutrophil development and the adenosine a2a receptor (*ADORA2A*; p < 10^−109^), which inhibits neutrophil degranulation; and the epigenetic regulators histone deacetylase 9 (*HDAC9*; p < 10^−4^; Supplementary Fig. S4c) and *DNMT3A* (p < 10^−46^). The highly significant hypermethylation within *DNMT3A* was further validated by bisulfite pyrosequencing in independent samples (Supplementary Fig. S3b). On the other hand, hypomethylated CpG sites were related to the transcription factor *PAX5* (p < 10^−5^) and recombination activating gene 2 (*RAG2*; p < 10^−8^), which are involved in lymphoid differentiation (Supplementary Fig. S4d). Gene Ontology (GO) analysis of hypermethylated genes revealed highly significant enrichment in functional categories of the immune system, hematopoietic development and activation (Fig. 2c), whereas classification of hypomethylated CpG sites was hardly significant and rather associated with signaling.

DNAm changes were then analyzed in the context of gene regions or CpG islands (CGIs; Fig. 2d). Culture associated DNA-hypermethylation was highly significantly enriched in upstream promoter-regions (TSS1500) and in 5′untranslated regions (UTRs). Furthermore, it was enriched in the 2 kb flanking regions of CGIs which are termed shore regions and which are strongly related to gene expression^19^. In contrast, hypomethylated CpG sites were rather enriched in 3′UTRs, intergenic regions, and more distant to CGIs. These results may indicate that particularly DNA-hypermethylation is functionally relevant.

**Figure 3:**
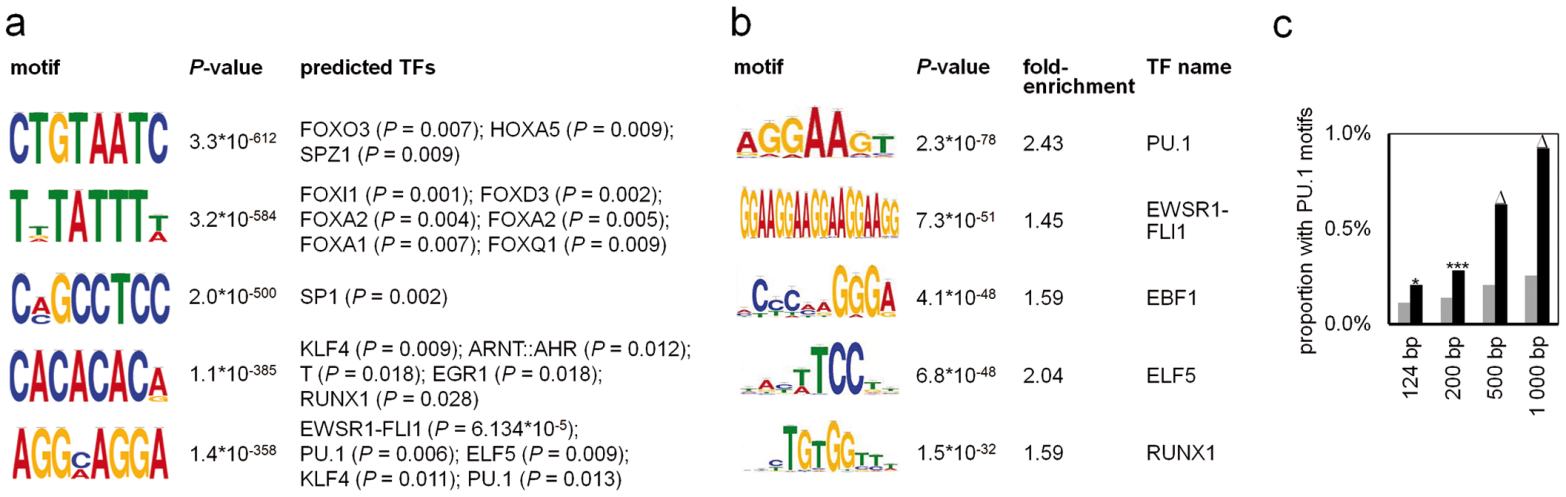
Enrichment of TF-binding motifs. (a) The sequences within 124 bp around each of the 9,213 overlappingly hypermethylated CpG sites were used for *de novo* motif analysis. The six most significant motifs were subsequently compared to known TF-binding sites. (b) Alternatively, these sequences were scanned for known motifs and enrichment and p-values (hypergeometric distribution) were calculated in relation to all CpG sites represented on the array. (c) Proportion of PU.1 binding sites within 124, 200, 500 and 1,000 bp around overlappingly hypermethylated CpG sites (black) was enriched compared to all CpG sites of the array (grey; *p < 0.05; ***p < 0.001; Δp < 10^−15^).

*De novo* motif discovery in the direct vicinity of hypermethylated CpG sites (124 bp) identified binding sites for forkhead box proteins, HOXA5, PU.1 and RUNX1 amongst several other transcription factors (TFs; Fig. 3a). Alternatively, we scanned for known TF-binding sites and the most significant results were observed for the TFs PU.1 (SPI1), EWSR1-FLI1, EBF1, ELF5, and RUNX1 (Fig. 3b). The importance of PU.1 binding sites is further supported by data of a ChIP-Seq experiment with CD133^+^ HPCs which were cultured for 10 days under very similar culture conditions (Fig. 3c)^20^.

**Figure 4:**
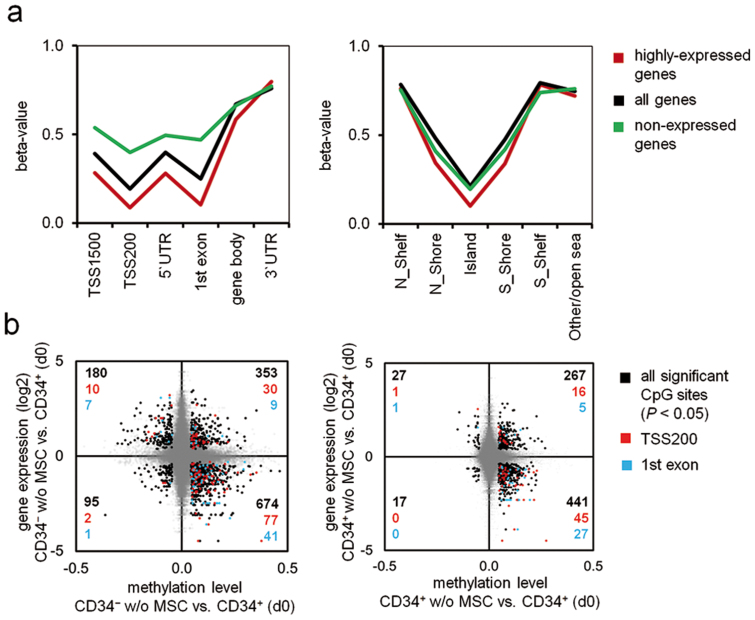
DNAm changes are reflected in differential gene expression. (a) Gene expression profiles were analyzed by microarray technology (n = 3). For 10% of the genes with highest (highly expressed genes) and 10% with least signal intensity (non-expressed genes) we determined average DNAm levels in different gene regions and in relation to CGIs. (b) Scatter plot analysis depicts CpG sites with differential DNAm (p < 0.05) and differential gene expression (p < 0.05). Higher methylation coincides with less gene expression, particularly in CpG sites located in TSS200 and 1^st^ Exon.

Subsequently, we analyzed gene expression profiles of CD34^+^ (d0), CD34^+^ w/o MSC and CD34^−^ w/o MSC. Co-cultured cells were omitted as gene expression is prone to highly expressed genes of contaminating MSCs (in contrast to DNAm profiles which are hardly influenced by the few additional DNA-strands)^21^. Signal intensity in microarrays was used as an indicator for the gene expression level: as expected, highly expressed genes (the top 10%) were hardly methylated at promoter regions, CGIs and shore-regions (Fig. 4a). Differential DNAm of all CpGs was then plotted against differential gene expression demonstrating that particularly hypermethylation in TSS200 and 1^st^ exon was associated with down-regulated gene expression (p < 10^−3^; Fig. 4b). In tendency, this association of high DNAm with low gene expression and *vice versa* became also evident by global analysis of all CpG sites although the correlation was only moderate.

**Figure 5:**
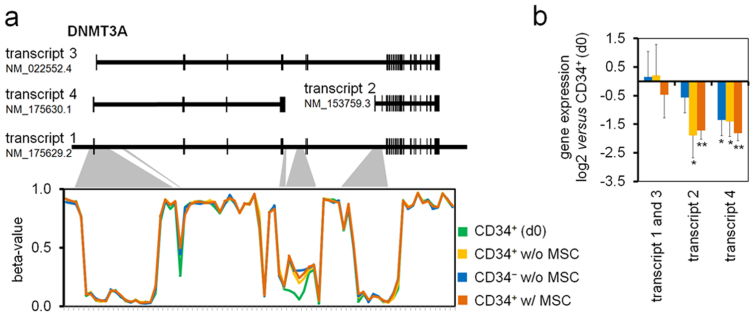
DNAm affects splice variants of DNMT3A. (a) DNAm is presented for CpG sites of *DNMT3A* and shaded areas depict localisation within the gene. Culture-associated hypermethylation is located at an internal promoter region between transcripts 2 and 4. (b) Quantitative RT-PCR analysis demonstrates that DNMT3A transcripts 2 and 4 are down-regulated upon *in vitro* expansion of HPCs (n = 5; *p ≤ 0.05; **p ≤ 0.01).

Hypermethylation of *DNMT3A* might be of specific relevance for induction of further DNAm changes – it was observed at an internal promoter region which did not affect overall gene expression but might influence expression of isoforms (Fig. 5a). In fact, RT-qPCR validated down-regulation of *DNMT3A* transcripts 2 and 4 upon culture expansion (Fig. 5b). In contrast, *DNMT3B* revealed neither differential DNAm nor differential gene expression.

### Inhibition of DNMTs modulates expansion of HPCs

**Figure 6:**
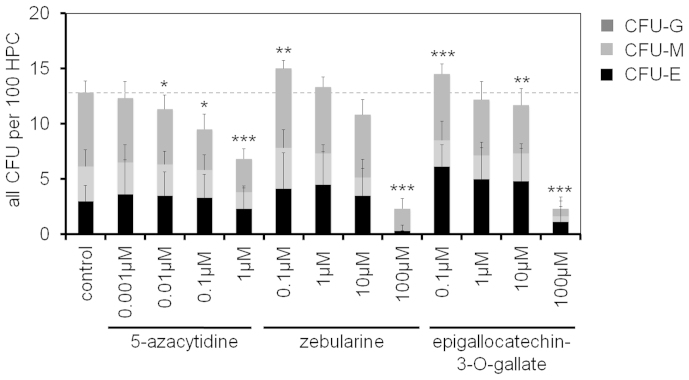
Treatment with demethylating agents increases CFU-frequency. HPCs were expanded with various concentrations of 5-azacytidine (AZA), zebularine (ZEB) and epigallocatechin-3-O-gallate (EGCG) and CFU frequency was analyzed after 7 days. Low concentrations of ZEB and EGCG increased CFU-frequency (n = 6; *p ≤ 0.05; **p ≤ 0.01; ***p ≤ 0.001). See also Supplementary Fig. S5.

To test the influence of demethylating agents on CFU frequency, we used the two cytidine analogues 5-azacytidine (AZA) and zebularine (ZEB), which can be incorporated into the DNA and then covalently bind to catalytic sites of DNMT1, DNMT3A and DNMT3B^22^. Alternatively, we used epigallocatechin-3-O-gallate (EGCG), which has also been shown to inhibit DNMTs^23^. Nano-molar doses of DNA-demethylating agents were shown to exert antitumor effects^24^ and therefore we tested a broad range of concentrations. Overall, proliferation and the percentage of CD34^+^ cells decreased with higher doses of inhibitors (Supplementary Fig. S5a,b). However, very low concentrations (0.1 μM) of either ZEB (p = 0.0077) or EGCG (p = 0.0009) slightly increased CFU-frequency suggesting enhanced progenitor expansion (Fig. 6).

## Discussion

In this study, we analyzed DNAm profiles of CD34^+^ cells and their progeny upon *in vitro* culture. We demonstrate that HPCs acquire highly reproducible DNAm changes within seven days *in vitro*. The faster proliferating progeny - which loses CD34 expression - reveals lineage-associated DNAm changes such as hypermethylation in the promoter regions of *CD34* and *CD133*. It was however unexpected, that also cells which maintained CD34^+^ demonstrated a similar extend of DNA-hypermethylation and this was hardly affected by stromal support. Previous studies have shown that normal hematopoietic differentiation is associated with hypomethylation of lineage specific genes^25^,^26^,^27^,^28^, and that DNA-methylation may protect from aberrant transcription factor activation^29^,^30^. In this regard, the culture-associated hypermethylation observed in this study does not seem to reflect normal hematopoietic differentiation but it rather resembles a roadblock for differentiation towards specific lineages.

It needs to be pointed out, that the CD34^+^ fraction is relatively heterogeneous and does not resemble pure stem cells. In fact, recent studies indicated that the most primitive HSCs are even CD34^−^31. In our previous work, we have demonstrated that the culture conditions used in this study maintain NOD/SCID repopulating cells^13^ – but we did not perform primary or secondary competition transplantation experiments to ultimately address preservation of long-term repopulating HSCs (LT-HSCs). Expression of the myeloid marker CD13 in many freshly isolated and culture expanded CD34^+^ cells supports the notion that our cell preparations comprise also many more differentiated progenitor cells – although this surface marker may also be expressed on primitive HSCs^32^. It is conceivable, that culture-associated DNAm changes differ in highly purified LT-HSCs. To this end, it is important to further optimize DNAm profiling technology for picogram DNA amounts obtained from a few thousand cells.

The prevailing DNA-hypermethylation in HPCs during *in vitro* culture implies that *de novo* methyltransferases are involved in this process. Conditional ablation of *Dnmt3a* in mice has been shown to increase the stem cell pool and to impair differentiation^33^. The relevance of DNMT3A is further supported by frequent mutations in acute myeloid leukemia (AML)^34^ and myelodysplastic syndromes (MDS)^35^. We have recently observed that aberrant DNA-hypermethylation frequently occurs within the gene *DNMT3A* in AML patients thereby mimicking genomic mutations in *DNMT3A* (Jost et al., in revision). Notably, in this study we describe that the same internal promoter region of *DNMT3A* is also hypermethylated in HPCs upon *in vitro* culture. This hypermethylation seems to be associated with down-regulation of the isoforms 2 and 4. Intragenic methylation has previously been shown to regulate alternative promotors^36^. Transcript 4 does not have methyltransferase activity but its down-regulation may still be functionally relevant if it contributes to regulative complexes. It is also intriguing that DNAm changes were enriched in *HOX* genes. The *HOXA* and *HOXB* clusters are relevant for hematopoietic development and their DNA-hypermethylation has been implicated in various hematopoietic malignancies^37^. Our analysis revealed that hypermethylated CpG sites are often associated with a HOXA5 binding motif and with binding sites for PU.1 and RUNX1. PU.1 together with GATA1 is involved in hematopoietic cell fate decisions^38^ and has been shown to interact with DNMT3A/B^39^. RUNX1 may interact with DNMT1^40^ for site specific DNAm. Simultaneous depletion of RUNX1 and the polycomb group protein PCGF1 allows sustained self-renewal while blocking differentiation of lineage marker-negative cells *in vitro*^41^. These findings support the notion that HOXA5, PU.1 and RUNX1 are involved in regulation of DNAm changes during culture of HPCs.

The results of this study point to distinct DNA-hypermethylation of HPCs during *in vitro* culture. Therefore, it appears to be obvious to test if this process can be blocked by DNAm inhibitors. In fact, low doses of AZA have previously been shown to support expansion of CD34^+^ cells *in vitro*^42^ and combination of AZA with trichostatin A supports maintenance of NOD/SCID mice repopulating cells^43^. We demonstrate that low doses of ZEB and EGCG facilitate a moderate but significant increase in CFU frequency. Several authors have analyzed the effect of DNMT inhibitors on DNAm profiles in normal HPCs and leukemic cells^24^,^44^. For example, Tsai and co-workers have analyzed DNAm profiles of CD34^+^ cells treated with or without 10 nM Decitabine^24^. This treatment resulted in an overall decrease of DNAm level with preference for specific genomic regions. However, when we compared DNAm changes in CD34^+^ cells upon treatment with this DNMT inhibitor and upon culture expansion there was no significant correlation (results not shown). This may be expected, as the mechanism of chromatin modifying agents is different and not site specific. To this end, a better understanding of the functional complexes which govern differential DNAm upon culture of HPCs is required. Direct targeting of these complexes might ultimately block functional changes in culture.

Taken together, *in vitro* culture of HPCs is associated with loss of differentiation potential in most of the progeny, even though the immunophenotypic characteristics may be maintained. Shared expression of CD34 – and of other surface markers - does not provide any reliable means of saying these cells are functionally similar. We demonstrate that *in vitro* culture of HPCs is associated with highly reproducible DNA-methylation changes. They are particularly observed in hematopoietic genes and we provide evidence that specific regulatory complexes, such as PU.1, RUNX1, HOXA5 and specific isoforms of DNMT3A, are involved in this process. These epigenetic modifications are reflected on gene expression level and they may interfere with subsequent differentiation. The specific epigenetic modifications may also be relevant for localisation of viral integration sites in the genome of CD34^+^ cells in gene therapy. Our experimental design does not allow conclusions on DNAm changes in purified HSCs – further research is required to determine whether the small subset of LT-HSCs undergoes epigenetic modifications *in vitro* such as CD34^+^ cells. However, the findings of this study indicate that *in vitro* manipulation of HPCs is associated with specific DNAm changes which seem to be associated with loss of differentiation potential.

## Methods

### Isolation of HPCs and culture conditions

CD34^+^ cells were isolated from fresh umbilical cord blood after written consent according to the guidelines specifically approved by the Ethic Committee of RWTH Aachen University (Permit Number: EK187/08) using the CD34 Micro Bead Kit on a MiniMACS system (Miltenyi Biotec GmbH, Bergisch-Gladbach, Germany). HPCs were cultured in StemSpan serum free expansion medium (STEMCELL Technologies, Grenoble, France) supplemented with 10 μg/mL heparin (Ratiopharm GmbH, Ulm, Germany), 20 ng/mL thrombopoietin (PeproTech GmbH, Hamburg, Germany), 10 ng/mL stem cell factor (PeproTech), 10 ng/mL fibroblast growth factor 1 (PeproTech). Culture was either performed on tissue culture plastic (TCP) or on a confluent layer of MSCs (passage 3 to 6). Mesenchymal stromal cells were cultured in Dulbecco's Modified Eagle's Medium-Low Glucose (PAA, Pasching, Austria) supplemented with 2 mM l-glutamine (Sigma, St Louis, MO, USA), 100 U/mL penicillin/streptomycin (Lonza, Basel, Switzerland), 10% pooled human platelet lysate^45^, and 2 U/mL heparin (Roche GmbH, Mannheim, Germany)^46^. Isolation and characterization of bone marrow derived MSCs was performed as described before^13^. After seven days, CD34^+^ and CD34^−^ fractions were again separated as described above. In some experiments, we added the DNMT-inhibitors 5-azacytidine (Sigma-Aldrich), zebularine (Enzo Life Sciences, Farmingdale, NY, USA) and epigallocatechin-3-O-gallate (Enzo Life Sciences) as indicated in Fig. 6.

### CFSE staining

Freshly isolated CD34^+^ cells were labelled with carboxyfluorescein diacetate N-succinimidyl ester (CFSE; Sigma-Aldrich, Steinheim, Germany) to monitor the number of cell divisions. In brief, CD34^+^ cells were washed with PBS and stained with CFSE at a final concentration of 2.5 μM in PBS with 0.1% fetal calf serum (FCS; PAA, Pasching, Austria) for 10 min at 37°C as described before^7^,^47^. To estimate fold-expansion rates of CD34^+^, we cultured 1,000 CD34^+^ cells with or without MSCs. After seven days, we analyzed the cell numbers with a CASY cell counter (Schärfe System, Reutlingen, Germany) and multiplied them with the percentage of CD34^+^ cells.

### Flow cytometric analysis

HPCs were stained with propidium iodide (PI), CD34-APC (Becton Dickinson, San Jose, CA, USA [BD], clone 581), CD133/2-PE (Miltenyi Biotec GmbH, clone AC141), CD45-V500 (BD, clone HI30), CD13-PE (BD, clone WM15), CD19-FITC (BD, clone HIB19), CD3-PerCP-Cy5.5 (BD, clone UCHT1), CD56-PE (BD, clone B159) with or without CFSE as indicated in the text. Cells were analyzed using a FACS Canto II (BD) running FACS Diva software (BD).

### Colony forming unit assays

10,000 CD34^+^ cells were cultured for one week as indicated in the text and subsequently re-seeded in different dilutions (1:10 and 1:100 dilution) in methylcellulose (HSC-CFU lite with EPO; Miltenyi Biotec)^13^. CD34^+^ (d0) cells were covered with methylcellulose directly after isolation. Granulocyte (CFU-G), macrophage (CFU-M) and erythrocyte colonies (CFU-E) were counted according to manufacturer's instructions.

### DNAm profiling

DNAm profiles were analyzed for three corresponding subsets of freshly isolated CD34^+^ cells (d0), CD34^+^ w/o MSC, CD34^−^ w/o MSC and CD34^+^ w/MSC. Genomic DNA was isolated from 10^6^ cells using the QIAamp DNA Blood Midi Kit (Qiagen, Hilden, Germany) and subsequently bisulfite-converted (EZ DNA Methylation™ Kit; Zymo, Irvine, USA). DNAm profiles were then analyzed with InfiniumHumanMethylation450 BeadChips (Illumina, San Diego, USA) according to the manufacturer's instructions. Hybridization and initial data analysis with the BeadStudio Methylation Module was performed at the DKFZ Gene Core Facility (Heidelberg, Germany). Raw data have been deposited at the public database Gene Expression Omnibus (GEO) under the accession number GSE40799.

For each of the 485 577 CpG sites, DNAm is provided as beta-values ranging from 0 (non-methylated) to 1 (100% methylation). Box plots and histograms were calculated in R. For statistical analysis, we confined 50% of the CpG sites according to variation in DNAm across different samples and calculated adjusted p-values using limma paired t-test in R (*P* < 0.05; adjusted for multiple testing). Affiliation of CpG sites to gene regions or CGIs was used as described in detail before^48^ and enrichment was determined by hypergeometric distribution. For graphical presentation of DNAm in specific genes, we compiled all CpG sites related to the corresponding genomic regions. For clarity reasons, the mean of three biological replica is presented although the inter-experimental variation was very small.

Gene Ontology classification of genes with either hypermethylated or hypomethylated CpG sites was performed with GoMiner software (http://discover.nci.nih.gov/gominer/). Enrichment of specific categories was estimated by one-sided Fisher's exact p-value using all CpG sites represented on the array as a reference. Alternatively, we used Gene Set Enrichment Analysis which provided similar results (GSEA; http://www.broadinstitute.org/gsea/index.jsp).

Motif analysis was performed with different programs of the MEME suit (http://meme.nbcr.net). De novo motif discovery was performed with DREME 3, an algorithm for identification of short, core DNA-binding motifs of transcription factors (TFs) within 124 bp around hypermethylated CpG sites. The most significant motifs were subsequently used for the TOMTOM motif comparison tool to identify potential TFs which bind to these motifs. Furthermore, we scanned for the occurrence of given motifs using the FIMO tool 4 within 124 bp around hypermethylated CpG sites (alternatively, we have extended these regions to 2 kb, but the high number of TF-binding sites made these results less specific). Enrichment was estimated in relation to all CpG sites represented on the array (hypergeometric distribution). Binding sites for PU.1, predicted by ChIP-Seq on cultured HPCs, were obtained from Novershtern et al^20^. Enrichment of PU.1 binding sites close to hypermethylated CpG sites was calculated by Fisher's exact test. We tested co-location window-values of 124, 200, 500 and 1,000 bp around hypermethylated CpG sites and used all CpGs in the array as background.

### DNAm analysis by bisulfite pyrosequencing

Genomic DNA was isolated and bisulfite-converted as described above. Specific regions within *CD34* and *DNMT3A* genes were then amplified by PCR and 20 μl of the product was immobilized to 5 μl Streptavidin beads followed by annealing to 0.8 μl sequencing primer (20 μM) for 2 min at 80°C. Primer information is provided in Supplementary table S1. Pyrosequencing was then performed on a PyroMark Q96 ID System and results were analyzed with the PyroMark Q CpG software (Qiagen).

### Gene expression analysis

Gene expression profiles were analyzed in three additional CB samples for CD34^+^ (d0), CD34^+^ w/o MSC and CD34^−^ w/o MSC. RNA was isolated from 10^6^ cells using the miRNeasy Mini Kit (Qiagen). Quality control and measurement of RNA concentration was done with a NanoDrop Spectrophotometer (Thermo Scientific, Wilmington, USA) and the Agilent 2100 Bioanalyzer (Agilent Technologies). Gene expression profiles were determined on GeneChip Human Gene 1.0 ST Arrays (Affymetrix, Santa Clara, CA, USA) according to the manufacturer's instructions. Raw data were normalized by RMA (Affymetrix Power Tools) and matched to DNAm data by gene ID. Data are accessible at GEO under GSE40669.

### RT-qPCR analysis of DNMT3A transcripts

Expression of DNMT3A transcripts was analyzed by real-time quantitative PCR (RT-qPCR) using the StepOneTM Instrument (Applied Biosystems, Applera Deutschland GmbH, Darmstadt, Germany). Total RNA (250 ng) was reverse-transcribed using the high capacity cDNA Reverse Transcription Kit (Applied Biosystems). cDNA was diluted 1:2 with nuclease-free water and 2 μl were amplified using Power SYBR Green PCR Master Mix (Applied Biosystems). Gene expression was normalized to GAPDH. The following primers (Metabion, Martinsried, Germany) were used for amplification of DNMT3A transcripts: Transcripts 1 and 3: Forward: 5′-ACCCTGCCTGAAGCCTCAAG-3′, Reverse: 5′-AAGGTGAGCCTCGGCATGG-3′; Transcript 2: Forward: 5′-GTGGATCGTAGCCTGAAAG-3′, Reverse: 5′-TGGTGGCATTCTTGTCC-3′; Transcript 4: Forward: 5′-AAGCGGGTGAGTCCTCAGC-3′, Reverse: 5′-CATATGCGCAGGCTGCATCC-3′.

### Statistics

All results are indicated with standard error of the mean (SEM). To estimate the probability of differences, we have adopted the two-sided Student's T-test. Probability value of p < 0.05 denoted statistical significance.

## Author Contributions

C.W., T.W., Q.L., B.D., I.C., M.Z. and W.W. designed research, analyzed and interpreted data; C.W., T.W. and Q.L. performed experiments; M.W., B.D. and M.Z. contributed vital reagents; C.W. and W.W. wrote the manuscript, and all authors provided input to the manuscript.

## Supplementary Material

Supplementary Information Supplementary Information
