# Supplementary material for: Hematopoietic Stem and Progenitor Cells Acquire Distinct DNA-Hypermethylation During in vitro Culture
Source: Sci Rep. 2013 Nov 28;3:3372. doi: 10.1038/srep03372 (PMC3842544; doi:10.1038/srep03372)
Supplement: Supplementary Information [file srep03372-s1.doc]

**Supplementary Information**

**Hematopoietic Stem and Progenitor Cells Acquire Distinct DNA-Hypermethylation During *in vitro* Culture.**

Carola Ingrid Weidner, Thomas Walenda, Qiong Lin, Monika Martina Wölfler, Bernd Denecke, Ivan Gesteira Costa, Martin Zenke, and Wolfgang Wagner

**
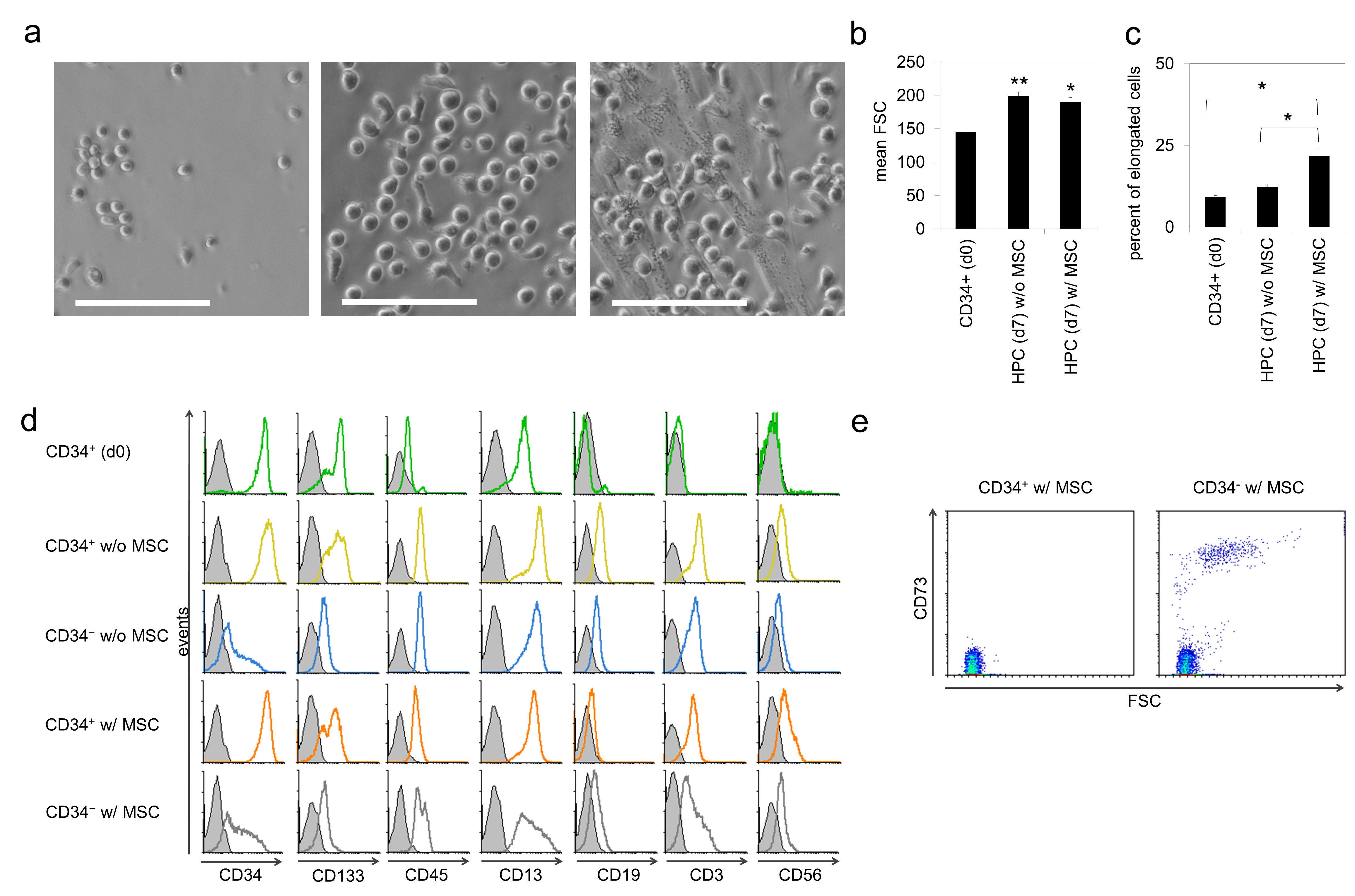
**

## Figure S1. Morphological and immunophenotypic changes upon culture of HPCs.

(**a**) Representative phase contrast images of freshly isolated CD34^+^ cells (left) and upon expansion without (middle) or with stromal support (MSCs; right). Scale bar: 100 µm. (**b**) Overall, the cell size increased upon culture and this is also reflected by forward scatter (FSC) analysis (n = 3; **P* ≤ 0.05; ***P* ≤ 0.01). (**c**) Cells were classified in either round or rather elongated morphology according to their appearance in phase contrast images (500 cells were considered per sample). The percentage of elongated cells increased particularly under co-culture conditions with MSCs (n = 3). (**d**) Freshly isolated (d0) CD34^+^ cells were cultured for seven days with or without MSCs, separated in CD34^+^ and CD34^−^ subsets (99.4% purity of CD34^+^ cells in the CD34^+^ w/o MSC and CD34^+^ w/ MSC samples) and then analyzed for CD34, CD133, CD45, CD13, CD19, CD3 and CD56 expression. This analysis demonstrates efficient separation of CD34^+^ and CD34^−^ fractions and moderate up-regulation of differentiation markers upon expansion. (**e**) Upon co-culture, MSCs are retained in the CD34^−^ fraction (CD34^−^ w/ MSC) – they can be distinguished from HPCs by larger cell size (forward scatter; FSC) and CD73 expression.


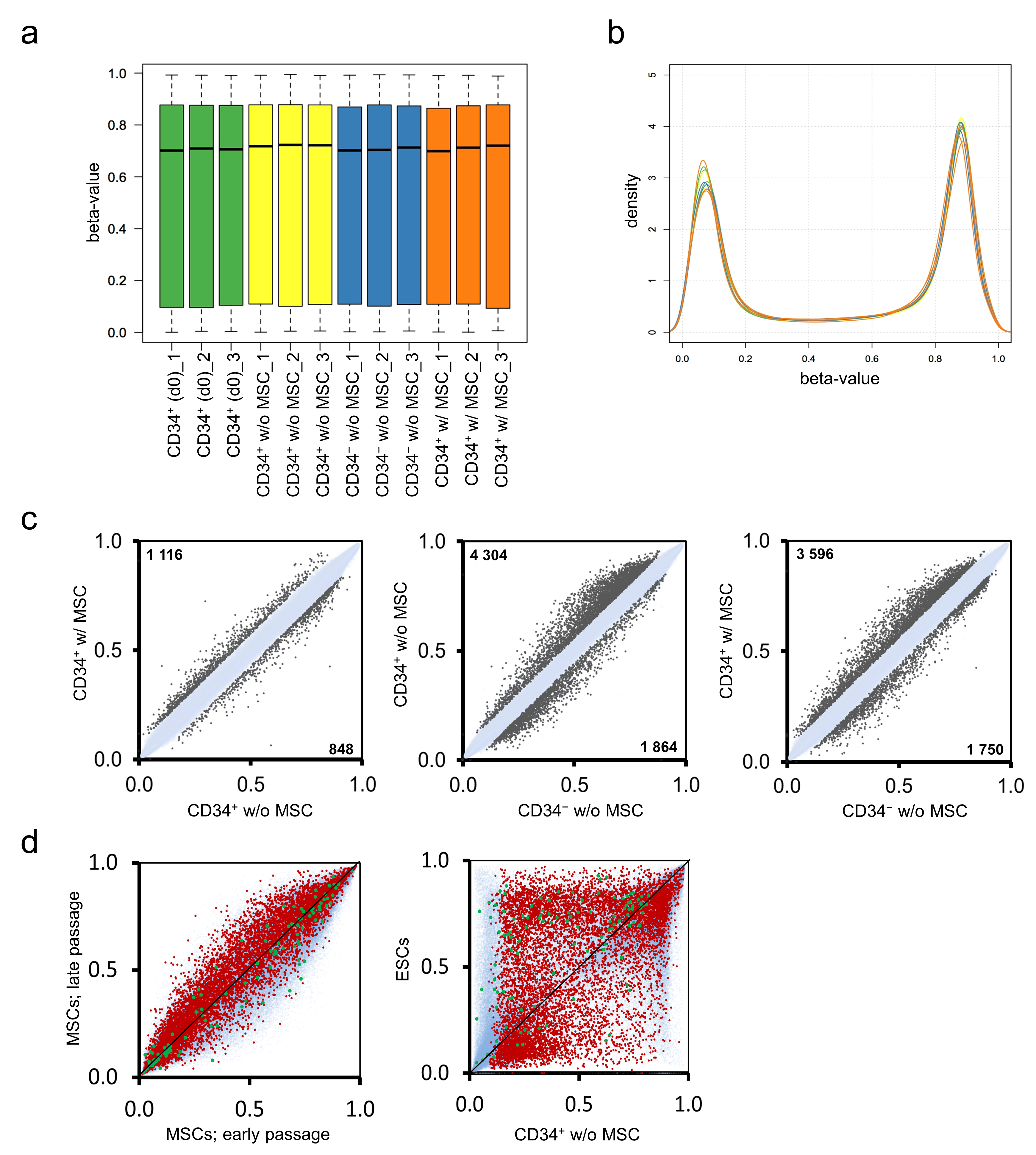


## Figure S2. DNAm changes during expansion of HPCs.

Box-plot (**a**) and histogram (**b**) of DNAm in more than 480 000 CpG sites for each sample analyzed on the Infinium HumanMethylation450 platform. Overall, the DNAm level was very similar in freshly isolated and expanded cells. (**c**) Comparison of DNAm profiles of CD34^+^ and CD34^−^ fractions after culture expansion. Grey dots represent significant hyper- or hypomethylation (adjusted *P*-value < 0.05) and the corresponding number of CpG sites is indicated. (**d**) Long-term culture has been shown to induce specific senescence-associated DNAm changes in MSCs (SA-DNAm).^1, 2^ More recently, we have demonstrated that SA-DNAm changes are reversed by reprogramming into induced pluripotent stem cells (iPSCs).^3^ Such analysis of SA-DNAm is hampered in CD34^+^ cells by the fact that they undergo functional and phenotypic changes during culture which may rather be associated with differentiation. We have therefore matched hypermethylated (red; 9 213 CpG sites) and hypomethylated (green; 113 CpG sites) CpG sites in HPCs with datasets of other cell types to estimate if they are associated with a similar process (GSE37066): comparison with DNAm profiles of MSCs at early and late passage^3^ revealed a moderate – but overall highly significant (*P* < 10^-100^) – association with SA-DNAm in MSCs. Unlike SA-DNAm changes the culture-associated DNAm changes in HPCs were not counteracted in pluripotent cells such as the embryonic stem cell line H1 (ESCs). These results indicate that DNAm changes in HPCs upon seven days expansion are hardly associated with senescence-associated DNAm changes which evolve during long-term culture.


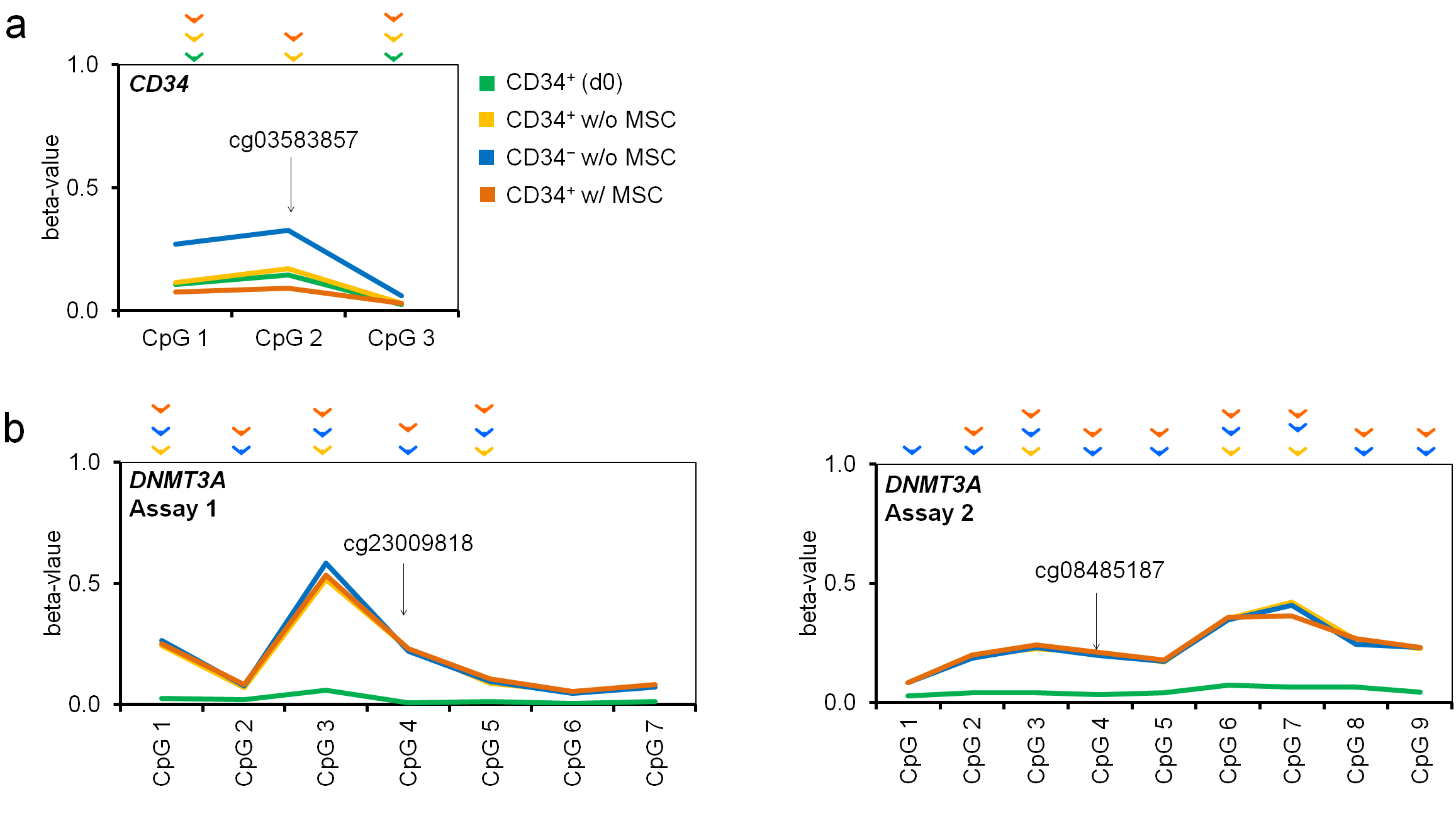


## Figure S3. Pyrosequencing validation of DNAm changes in *CD34* and *DNMT3A.*

Differential DNAm was exemplarily validated in independent cell preparations for CpGs associated with the promoter of *CD34* or the internal promoter region of *DNMT3A*. (**a**) For *CD34* the DNAm was always higher in the CD34^-^ subset – in analogy to findings of the 450k Illumina BeadChip as depicted in Figure 1g. Arrowheads represent significance as compared to CD34^-^ w/o MSCs (p < 0.05; two-sided paired t-test). (**b**) DNAm in *DNMT3A* was analyzed with two different pyrosequencing assays which resemble seven and nine CpG sites in close vicinity. DNAm was always higher in culture-expanded subsets as compared to freshly isolated cells. Arrowheads indicate significance as compared to CD34^+^ (d0) (p < 0.05). These results are consistent with figure 5a. CpG sites represented on the 450k BeadChip are indicated (cg0353857, cg23009818 and cg08485187).


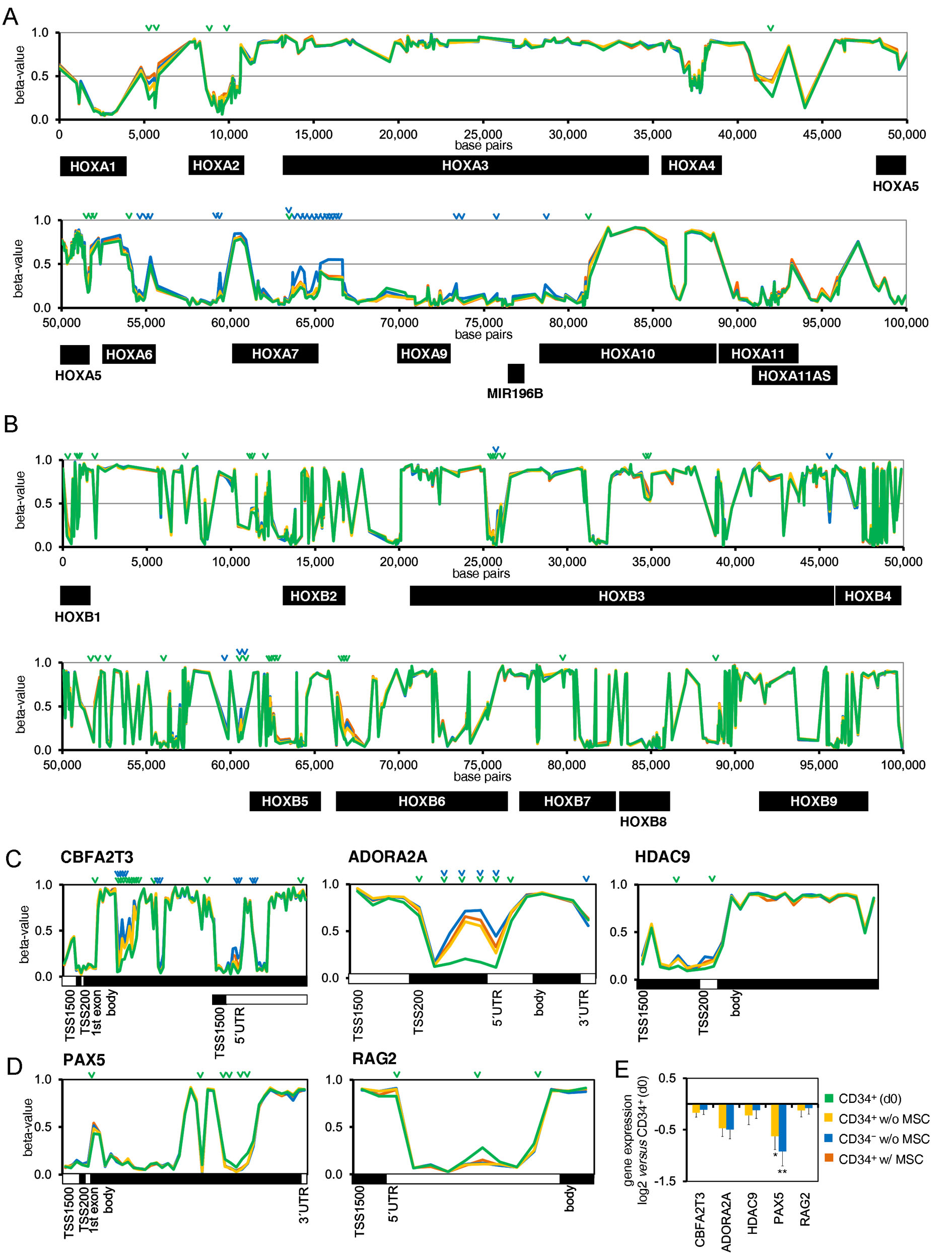

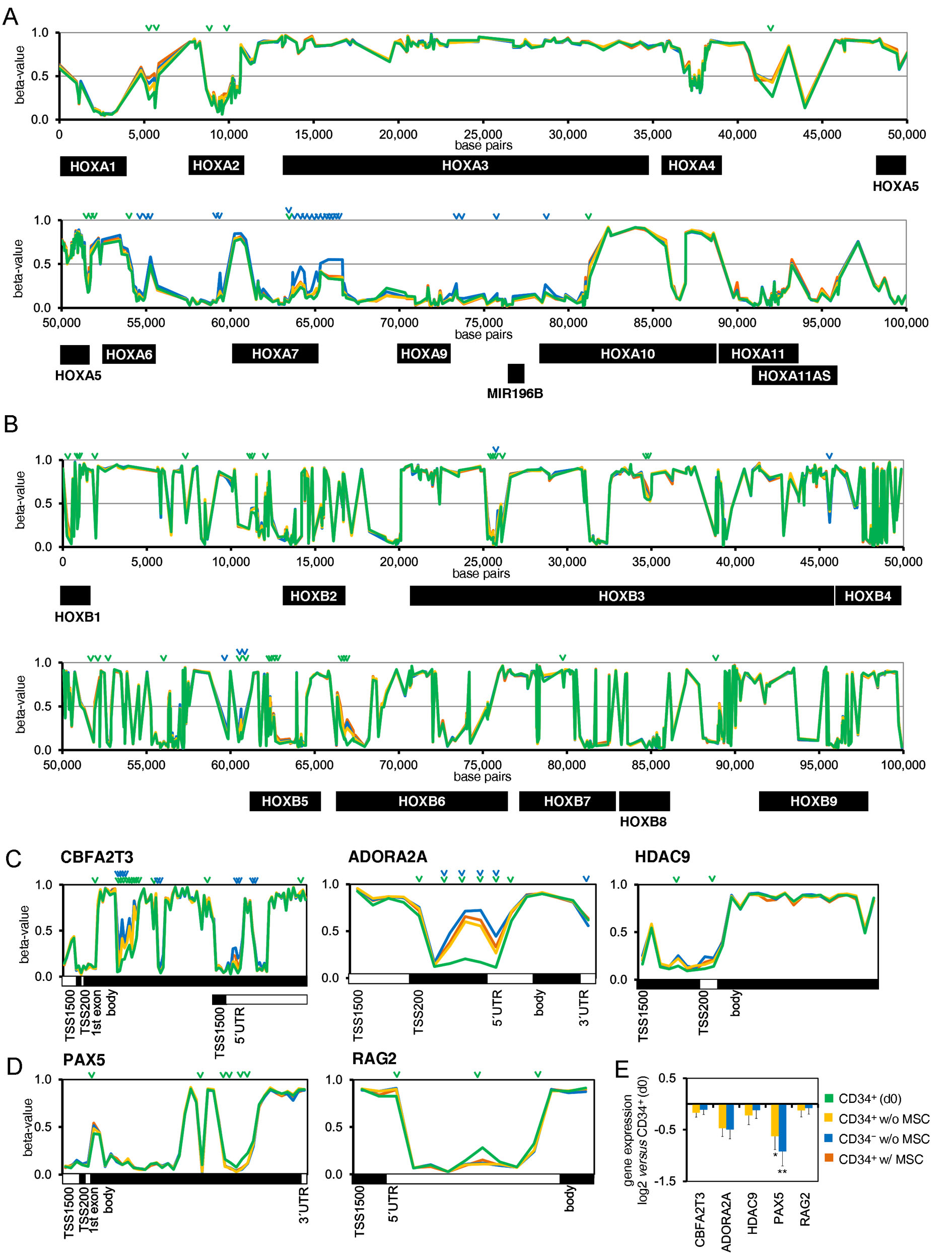

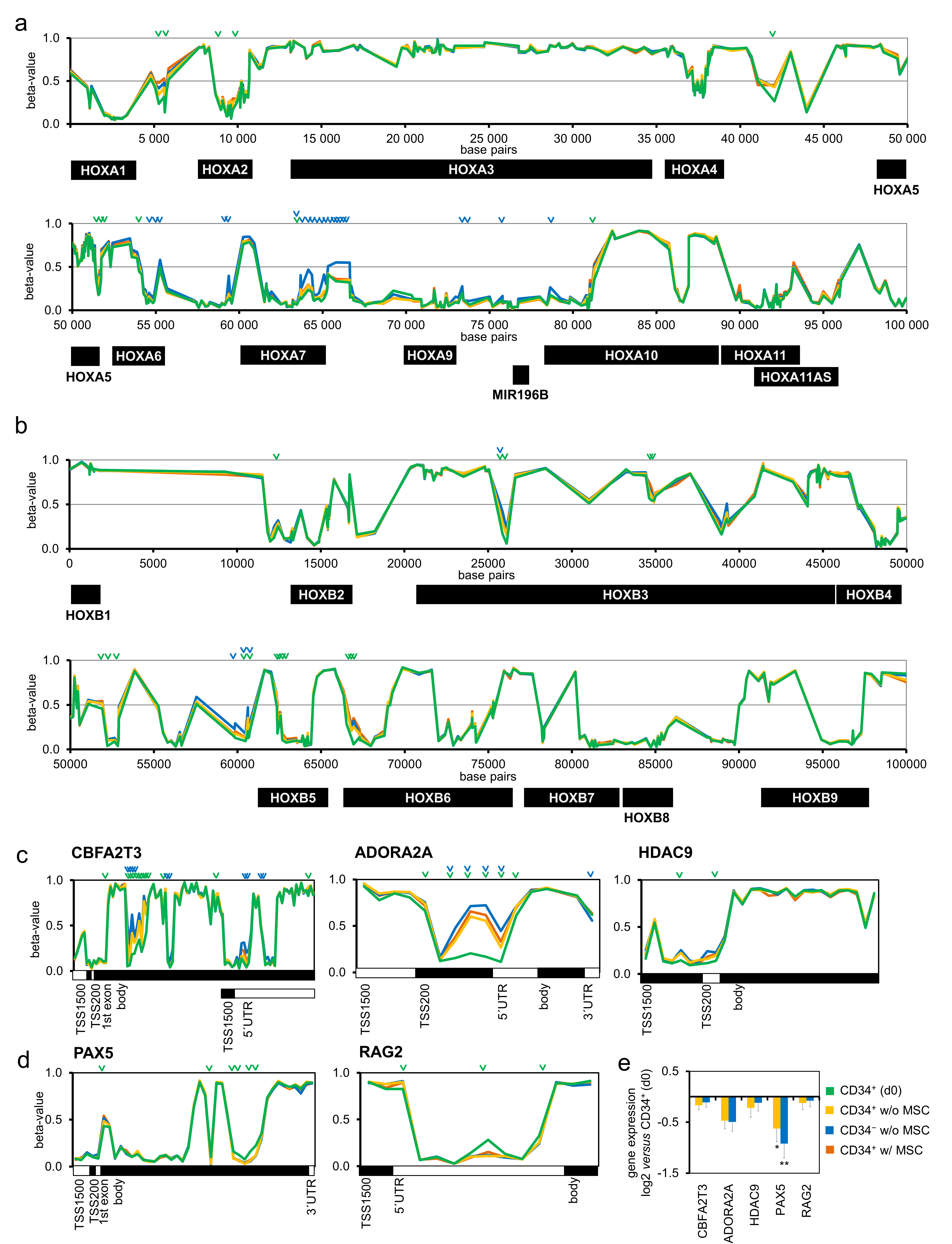


## Figure S4. DNAm in selected genes.

DNAm pattern in the homeobox gene cluster A (**a**) and B (**b**; HOXA13 and HOXB13 are not shown). For each CpG site, the mean DNAm (beta-value) is presented for freshly isolated (green), and expanded subsets (CD34^+^ w/o MSC: yellow; CD34^−^ w/o MSC: blue; CD34^+^ w/ MSC: orange). CpG sites with significant DNAm changes (adjusted *P* < 0.05) are indicated by arrowheads (comparison to freshly isolated CD34^+^ cells depicted in green; comparison to CD34^−^ w/o MSC in blue). (**c**) DNAm of *CBFA2T3*, *ADORA2A* and *HDAC9.* Promoter-associated CpG sites become significantly hypermethylated upon culture. (**d**) The transcription factors *PAX5* and *RAG2,* which are involved in lymphoid differentiation, reveal significant DNA-hypomethylation. (**e**) Gene expression analysis (Affymetrix Gene ST 1.0 Array; n = 3) depicts down-regulation of the selected genes.


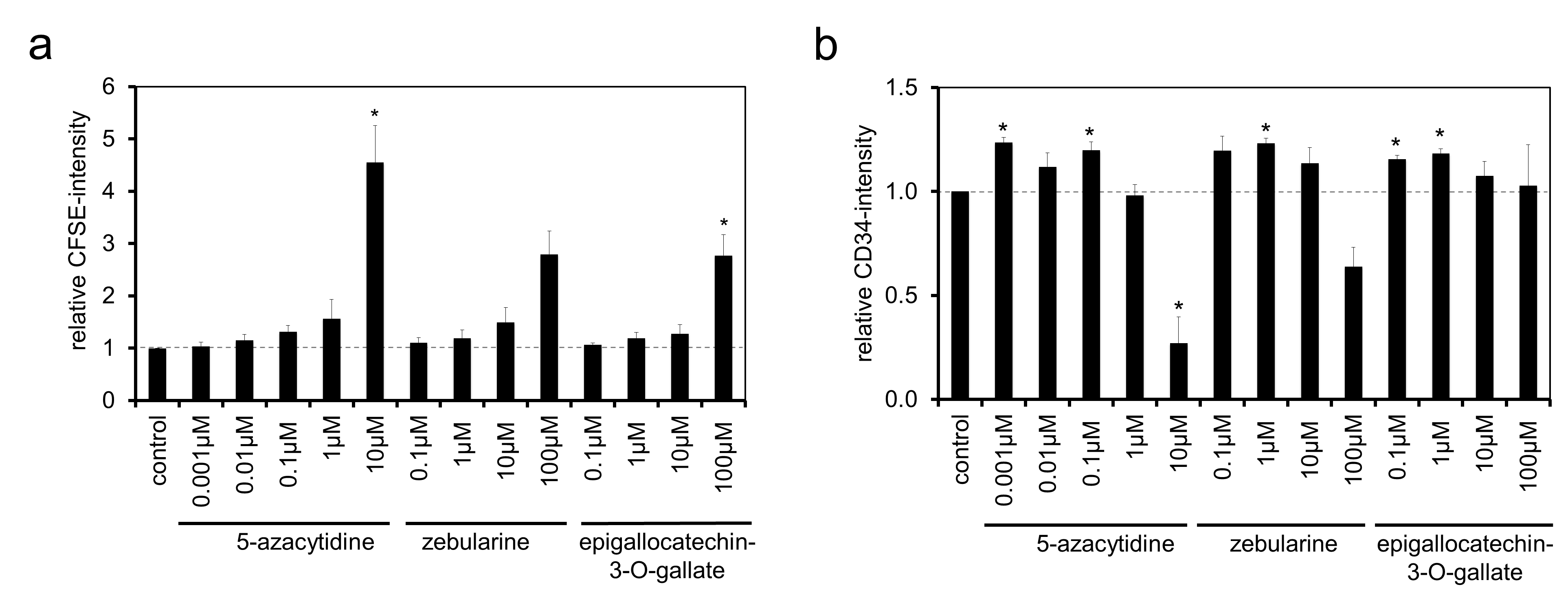


## Figure S5. DNMT-inhibitors affect proliferation of HPCs.

(**a**) CD34^+^ cells were stained with CFSE and then cultured for 7 days with various concentrations of 5-azacytidine (AZA), zebularine (ZEB) or epigallocatechin-3-O-gallate (EGCG). High concentrations impaired cellular proliferation (corresponding to more residual CFSE staining; n = 3). (**b**) The mean signal intensity of CD34 was increased upon treatment with low concentrations of AZA, ZEB, and EGCG (n = 3; **P* ≤ 0.05).

## Supplementary Table S1: Primer for pyrosequencing analysis

| **Primer** | **Sequence** |
| --- | --- |
| DNMT3A assay 1 forward | 5`-GGTTTGGGTTTATTGTAGGAAGGTTATTAAGGT-3´ |
| DNMT3A assay 1 reverse | 5`-Bio-AATCCAAAACCCCCCTATCACGAAA-3´ |
| DNMT3A assay 1 sequencing | 5`-GTGGGGGGAGATAAA-3´ |
| DNMT3A assay 2 forward | 5`-Bio-TTAGGTTCGGGAGTATTAGGGGGAGG-3´ |
| DNMT3A assay 2 reverse | 5`-AACAAAACCAAACAAAATAAATAAAACAACCC-3´ |
| DNMT3A assay 2 sequencing | 5`-AAATAAAACAACCCTTAAAA-3´ |
| CD34 forward | 5`-Bio-GGAATTTATTGTAAGGATTTTAGTTTAGAAGTG-3´ |
| CD34 reverse | 5`-AAAAAAATCTCCAAAAATAATTTAAACAACCA-3´ |
| CD34 sequencing | 5`-AACCAAAATAAATACTTATAATCA-3´ |

## References for Supplementary Material

# 1. Koch,C.M. *et al.* Monitoring of Cellular Senescence by DNA-Methylation at Specific CpG sites. *Aging Cell* 11, 366-369 (2012).

# 2. Schellenberg,A. *et al.* Replicative senescence of mesenchymal stem cells causes DNA-methylation changes which correlate with repressive histone marks. *Aging (Albany NY)* 3, 873-888 (2011).

# 3. Koch,C.M. *et al.* Pluripotent stem cells escape from senescence-associated DNA methylation changes. *Genome Res.* 23, 248-259 (2013).
